# Spheno-Orbital Meningiomas: A Systematic Review of Treatment Modalities, Adjuvant Therapies, and Recurrence Risk

**DOI:** 10.7759/cureus.73908

**Published:** 2024-11-18

**Authors:** Injam Ibrahim Sulaiman, Ali Hassan baker, Azhin Shafeeq, Mustafa Ismail

**Affiliations:** 1 Department of Surgery, Hawler Medical University, College of Medicine, Erbil, IRQ; 2 Department of Neurosurgery, Hawler Teaching Hospital, Erbil, IRQ; 3 Department of Surgery, Baghdad Teaching Hospital, Medical City Complex, Baghdad, IRQ

**Keywords:** adjuvant radiotherapy, minimally invasive surgery, orbital meningioma, proptosis, surgical resection

## Abstract

Spheno-orbital meningiomas (SOMs) are rare tumors that involve the sphenoid wing and orbit, leading to symptoms such as proptosis and vision loss. Their proximity to critical neurovascular structures complicates surgical resection, making management challenging. A systematic review of 22 paper series involving 1042 patients was conducted using PubMed and Scopus. Studies focused on SOM diagnosis, surgical techniques, recurrence rates, and the role of adjuvant therapies were analyzed. Proptosis and visual impairment were the most common symptoms. Gross total resection (GTR) reduced recurrence, but subtotal resection (STR) often led to higher recurrence, particularly in optic canal involvement. Minimally invasive approaches showed the potential to reduce morbidity. Adjuvant radiotherapy was effective in controlling tumor growth post-STR. SOM management requires a balance between tumor control and functional preservation. While GTR minimizes recurrence, STR with adjuvant radiotherapy is a viable alternative in challenging cases. Minimally invasive techniques offer promise, but further long-term studies are needed.

## Introduction and background

Spheno-orbital meningiomas (SOMs) are a rare subset of intracranial tumors, comprising approximately 9-18% of all meningiomas [1]. These tumors are notable for their unique location at the skull base, typically involving the sphenoid wing, and their characteristic invasion of the orbit, often leading to clinical presentations such as proptosis and visual impairments [2]. SOMs are histologically distinct due to their en plaque growth pattern, accompanied by hyperostosis, which further complicates their diagnosis and surgical management [1,2].

Although SOMs are generally benign, their complex anatomical location adjacent to critical neurovascular structures, such as the optic nerve, superior orbital fissure, and orbital apex, poses significant surgical challenges [3]. Gross total resection (GTR) is often pursued to minimize recurrence rates; however, achieving complete resection is frequently difficult due to the risk of damaging surrounding critical structures, which can lead to severe neurological deficits [3]. The management of SOMs has employed a variety of surgical approaches, such as frontotemporal craniotomies, optic canal decompression, and endoscopic techniques, but the balance between tumor removal and functional preservation remains a subject of debate [1].

Recurrence rates for SOMs vary widely, ranging from 0% to as high as 56%, depending on factors such as the extent of resection and the degree of involvement of critical structures, such as the optic canal, sphenoid bone, and superior orbital fissure [3]. When recurrence occurs, reoperation and, in some cases, adjuvant radiotherapy are necessary to control tumor progression and stabilize clinical symptoms [3].

This systematic review aims to evaluate the current literature on spheno-orbital meningiomas, focusing on recurrence patterns, risk factors, and the outcomes of different surgical techniques. By analyzing various treatment modalities, this review seeks to clarify the optimal strategies for improving surgical success and long-term disease control.

## Review

Method

Search Strategy

**Figure 1:**
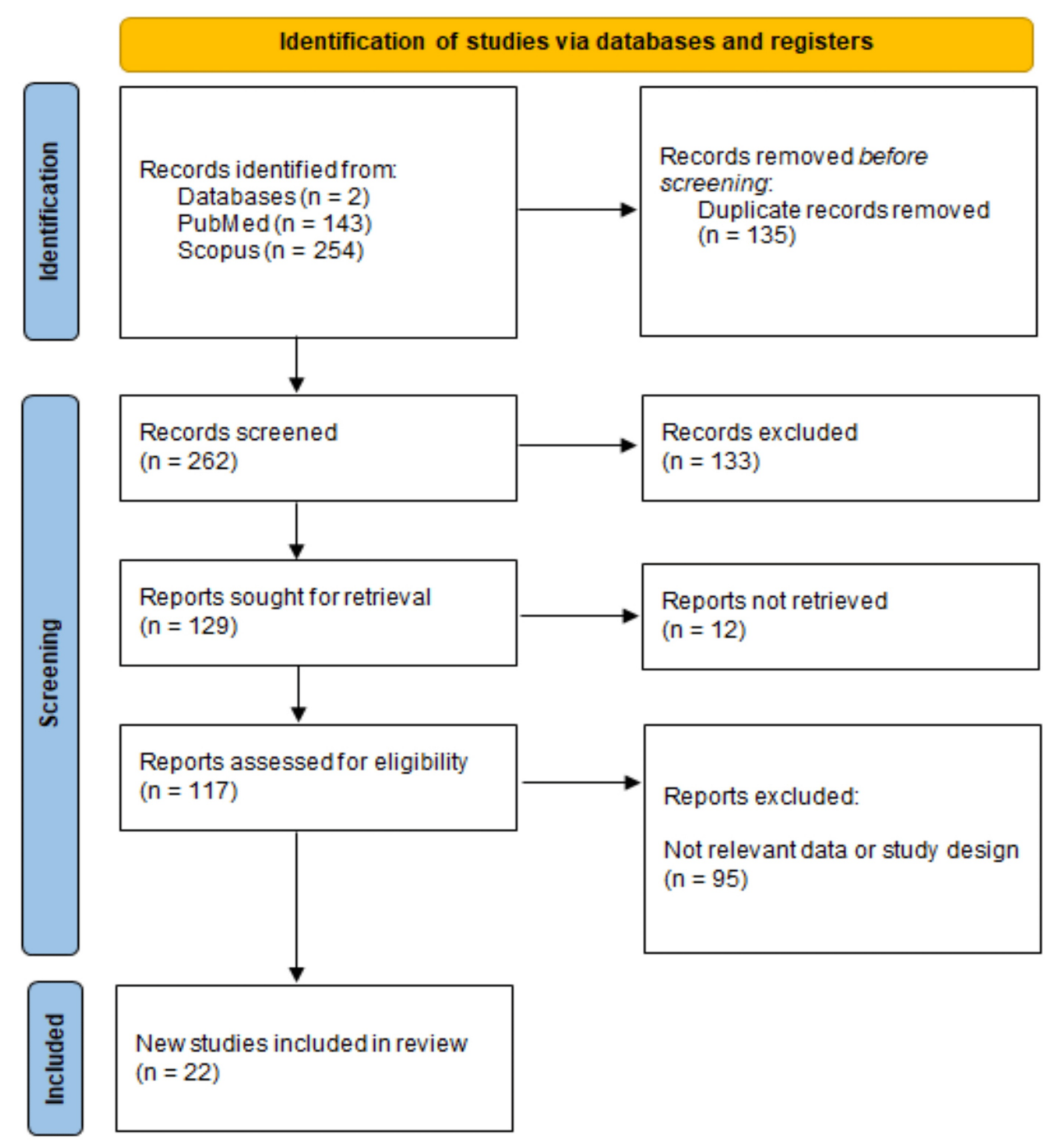
PRISMA flowchart of the included studies.

This systematic review was conducted following the Preferred Reporting Items for Systematic Reviews and Meta-Analyses (PRISMA) guidelines (Figure 1) [4]. A comprehensive literature search was performed using Scopus and PubMed databases to identify relevant studies published in English. The search strategy used a combination of Medical Subject Headings (MeSH) and keywords: ("Orbital Meningioma") AND ("Diagnosis" OR "Management" OR "Surgery" OR "Radiotherapy" OR "Prognosis").

Eligibility Criteria

Studies were eligible for inclusion if they focused specifically on orbital meningiomas and addressed aspects of diagnosis, management, surgical interventions, radiotherapy, or prognosis. Additionally, only original research articles were included, with review articles and case reports excluded. The articles needed to be published in English, with no restrictions on the publication date. Studies that did not explicitly address orbital meningiomas, such as those discussing other forms of meningiomas, were excluded from the review.

Study Selection

The process of selecting studies involved using Rayyan, a web-based tool for systematic reviews. After removing duplicates, two independent reviewers screened the titles and abstracts of the identified studies to assess their relevance. Any disagreements between the reviewers were resolved through discussion or, if necessary, by consulting a third reviewer. We retrieved the full texts of potentially eligible studies and further evaluated them against the predetermined eligibility criteria for final inclusion.

Data Extraction

Data extraction was performed using a standardized data collection form. The extracted data included details on study design, population characteristics, sample size, diagnostic methods, surgical techniques, adjuvant treatments such as radiotherapy, clinical outcomes, complications, and recurrence rates. Additionally, we recorded prognostic factors where reported.

Quality Assessment

The quality of the included studies was assessed using the ROBINS-I tool (Risk of Bias in Non-randomized Studies of Interventions) [5]. The tool was used to evaluate bias across seven domains: confounding, participant selection, classification of interventions, deviations from intended interventions, missing data, measurement of outcomes, and reporting bias. Each study was rated for risk of bias in these areas, and an overall judgment of low, moderate, or high risk of bias was made based on these criteria (Table 1) [3,6-26].

**Table 1:** ROBINS-I assessment of the included studies.

Study	Confounding	Selection of patients	Classification of interventions	Deviations from intended interventions	Missing data	Measurement of outcomes	Selection of reported results
Mariniello et al. [3]	Moderate	Low	Low	Moderate	Low	Moderate	Moderate
Sandalcioglu et al. [6]	Moderate	Low	Moderate	Moderate	Low	Moderate	Moderate
Najafabadi et al. [7]	Moderate	Low	Low	Moderate	Low	Moderate	Moderate
Nagahama et al. [8]	Moderate	Low	Low	Low	Moderate	Moderate	Low
Kong et al. [9]	Moderate	Low	Moderate	Moderate	Low	Moderate	Moderate
Furdová et al. [10]	Low	Moderate	Low	Low	Low	Moderate	Low
Mariniello et al. [11]	Moderate	Low	Low	Moderate	Low	Moderate	Moderate
Mariniello et al. [12]	Low	Low	Low	Moderate	Low	Moderate	Low
Lekovic et al. [13]	Low	Low	Low	Moderate	Low	Moderate	Low
Dallan et al. [14]	Moderate	Low	Low	Moderate	Low	Moderate	Low
Young et al. [15]	Moderate	Low	Low	Moderate	Low	Moderate	Moderate
Freeman et al. [16]	Moderate	Low	Low	Moderate	Low	Moderate	Moderate
Shapey et al. [17]	Moderate	Low	Moderate	Moderate	Low	Moderate	Low
Agi et al. [18]	Low	Low	Low	Moderate	Low	Moderate	Low
Terrier et al. [19]	Moderate	Low	Moderate	Low	Low	Moderate	Low
Mourits et al. [20]	Moderate	Low	Low	Moderate	Moderate	Low	Moderate
Terpolilli et al. [21]	Low	Low	Moderate	Low	Low	Moderate	Moderate
Cannon et al. [22]	Moderate	Low	Moderate	Moderate	Low	Moderate	Moderate
Honig et al. [23]	Moderate	Low	Moderate	Moderate	Low	Moderate	Moderate
Schick et al. [24]	Low	Low	Low	Moderate	Low	Moderate	Low
Schick et al. [25]	Moderate	Low	Moderate	Moderate	Low	Moderate	Moderate
Liu et al. [26]	Low	Low	Low	Moderate	Low	Moderate	Moderate

Data Synthesis

The findings from the included studies were synthesized narratively, and where quantitative data were available, a meta-analysis was conducted. The meta-analysis focused on the outcomes related to surgical interventions, recurrence rates, and the effectiveness of various treatment modalities. Subgroup analyses were performed based on factors such as the extent of tumor resection, type of surgical approach, and the use of adjuvant therapies.

Results

Study Characteristics

This systematic review synthesized data from 22 case series encompassing 1042 patients with SOMs, a complex subtype of meningiomas typically located at the skull base (Table 2) [3,6-26]. The majority of these studies were conducted in Europe, North America, and Asia, with sample sizes ranging from 4 to 130 patients. Across the studies, the average patient age ranged from 37 to 76 years, with a notable female predominance, consistent with the known epidemiology of meningiomas. Female patients accounted for 68-92% of cases, which reflects the recognized hormonal influences in meningioma pathogenesis.

**Table 2:** Summary of surgical and adjuvant therapy outcomes in spheno-orbital meningioma. CT: computed tomography; FTOZ: fronto-temporal orbito-zygomatic (surgical approach); MRI: magnetic resonance imaging.

Study	Design	Sample size	Country	Demographics	Location	Symptoms	Imaging	Treatment modalities	Adjuvant therapy	Complications	Recurrence	Follow-Up
Mariniello et al. [3]	Case Series	80	Italy	Age: 26–75, 82.5% female	Spheno-orbital	Proptosis, visual dysfunction	MRI, CT: optic canal involvement	Supraorbital-pterional, FTOZ for diffuse forms	12 received radiotherapy	Optic nerve damage, eye motility deficits	37.5%	Median: 136 months
Sandalcioglu et al. [6]	Case Series	16	Germany	Age: 37–76, mostly female	Spheno-orbital	Proptosis, visual loss, pain	MRI, CT: Bone and soft tissue invasion	Fronto-temporal craniotomy, orbitotomy, bone graft	2 cases with radiotherapy	Oculomotor palsy, meningitis, osteomyelitis	9 cases	Mean: 68 months
Najafabadi et al. [7]	Case Series	19	Netherlands	Median age: 47, 95% female	Spheno-orbital	Proptosis, visual loss, hyperostosis	CT, MRI: Hyperostosis	Pterional approach, decompression, implant	Proton therapy for 2 patients	Hypesthesia, facial nerve deficit, cellulitis	21% required reoperation	Median: 2.4 years
Nagahama et al. [8]	Case Series	13	Japan	Age: 20–71, 8 female, 5 male	Spheno-orbital	Proptosis, visual disturbance	CT, MRI: hyperostosis	Fronto-temporal craniotomy, optic canal decompression	Radiotherapy for WHO-II tumors	Diplopia, cerebral infarction, ptosis	4 cases	Mean: 74.4 months
Kong et al. [9]	Case Series	41	South Korea	Age: 24–73, 36 female	Spheno-orbital	Proptosis, visual impairment	MRI: hyperostosis, tumor size	Endoscopic transorbital approach, osteotomy	Gamma Knife radiosurgery	CSF leaks, keloid scar, infection	7 cases	Mean: 15.9 months
Furdová et al. [10]	Case Series	15	Slovakia	Mean age: 58.3, 73.3% female	Optic nerve, orbit	Protrusion, visual loss	MRI: brain and orbit	Enucleation, partial exenteration in 3 cases	Radiotherapy for progressive cases	None reported	No specific rate mentioned	Mean: 5 years

Clinical Presentation

Proptosis and visual impairment were the most commonly reported symptoms, consistent with the orbital and skull base involvement of these tumors. Proptosis was documented in the majority of patients across all studies, with some series, such as those by Mariniello et al. [3] and Terrier et al. [19], reporting very high rates. Visual impairment, ranging from mild visual field defects to severe visual loss, was frequently observed, with more severe visual loss noted in patients presenting later in the disease course. Diplopia and ocular motility disturbances were also common, particularly in cases involving the optic canal and superior orbital fissure.

Imaging and Diagnosis

Magnetic resonance imaging (MRI) and computed tomography (CT) were utilized in all studies to characterize the extent of both osseous and soft tissue involvement. Hyperostosis of the sphenoid wing and orbital invasion were hallmark features of SOMs, often leading to significant orbital remodeling and compression of the optic nerve. Hyperostosis was particularly well documented in studies by Najafabadi et al. [7] and Kong et al. [9], where CT imaging demonstrated its direct impact on both cosmetic and functional outcomes. Optic canal involvement noted in multiple studies, such as those by Mariniello et al. [3] and Freeman et al. [16], was identified as a critical factor influencing surgical planning and prognosis due to its impact on vision preservation.

Surgical Interventions

The primary treatment modality for SOMs across all studies was surgical resection, with the goal of maximally safe tumor removal. The most common approach was a fronto-temporal craniotomy, often combined with an orbitotomy or optic canal decompression, particularly in cases with optic nerve involvement. Studies like those by Mariniello et al. [12] and Young et al. [15] underscored the importance of decompression in preserving or restoring visual function. Minimally invasive approaches, including the endoscopic transorbital approach, were explored in more recent studies, such as those by Kong et al. [9] and Dallan et al. [14]. These approaches showed promise in reducing surgical morbidity while maintaining adequate resection rates, especially for tumors with anterior orbital involvement.

Adjuvant Therapy

Radiotherapy was employed in cases where GTR could not be achieved or in the presence of atypical (WHO grade II) tumors. Studies by Mariniello et al. [3] and Freeman et al. [16] demonstrated the utility of postoperative radiotherapy in reducing recurrence rates in patients with subtotal resections or higher-grade meningiomas. Gamma knife radiosurgery and proton therapy were reported as effective adjuncts in selected cases of recurrence, as noted by Kong et al. [9] and Lekovic et al. [13]. These treatments offered favorable control rates for residual tumor growth, particularly in anatomically challenging areas like the optic canal and cavernous sinus.

Complications

Complication rates varied considerably across the studies, largely depending on the extent of resection and the surgical approach used. Common complications included cranial nerve deficits, such as oculomotor palsy, and cerebrospinal fluid (CSF) leaks, which were reported in up to 30% of cases in series such as those by Sandalcioglu et al. [6] and Young et al. [15]. Diplopia and ptosis were also frequently noted, especially in cases requiring optic nerve decompression or extensive orbital manipulation. Infection rates, including cellulitis and meningitis, were reported but were relatively infrequent, occurring in <5% of cases in most studies. The newer endoscopic approaches appeared to reduce the incidence of such complications, although long-term follow-up data are still limited.

Prognosis and Outcomes

Surgical resection provided symptomatic relief in most patients, particularly in reducing proptosis and improving or stabilizing vision. Studies by Sandalcioglu et al. [6] and Agi et al. [18] showed that proptosis improved in up to 64.3% of patients, with many reporting stable or improved visual outcomes following surgery. However, patients presenting with severe preoperative visual deficits were less likely to experience significant visual improvement, a finding consistent across multiple studies. Early surgical intervention, particularly with optic canal decompression, was strongly associated with better visual outcomes, underscoring the need for timely diagnosis and management.

Recurrence

The extent of surgical resection is the most significant predictor of recurrence in SOMs. In most studies, gross total resection - where the tumor, including the hyperostotic bone and soft tissue components, is completely removed - was associated with the lowest recurrence rates. For example, studies by Mariniello et al. [3] and Freeman et al. [16] demonstrated that STR significantly increased the risk of recurrence, with reported recurrence rates of 37.5% and 48%, respectively, compared to much lower rates observed after GTR. This is consistent with the fact that residual tumors, particularly when involving critical structures like the optic canal or cavernous sinus, remain prone to regrowth over time. However, achieving GTR in SOMs is often constrained by the need to preserve critical visual and motor functions. Aggressive resection, especially when involving the optic canal, can carry a significant risk of morbidity, including vision loss and cranial nerve deficits. Studies such as those by Young et al. [15] and Sandalcioglu et al. [6] emphasize that careful balance must be maintained between the goals of maximal resection and functional preservation, especially in cases with extensive bone and soft tissue invasion. As a result, many surgeons favor a strategy of subtotal resection in cases where critical structures are involved, recognizing the potential for recurrence but prioritizing the avoidance of severe morbidity.

The choice of surgical approach can also influence recurrence rates. Traditional fronto-temporal craniotomies remain the standard for most surgeons due to their access to both the skull base and orbit, allowing for extensive resection of both the bony and soft tissue components of the tumor. However, newer approaches, such as the endoscopic transorbital approach, have gained attention for their potential to reduce morbidity while still allowing for substantial tumor debulking. As demonstrated by Kong et al. [9], this approach may reduce the extent of bony removal required, thus decreasing the risk of postoperative complications such as CSF leaks or cranial nerve injury while still offering satisfactory control of tumor progression. However, the long-term impact of these minimally invasive techniques on recurrence rates remains to be fully understood, as the follow-up data are still relatively short compared to more traditional methods.

Role of Neoadjuvant and Adjuvant Radiotherapy

For cases where gross total resection is not feasible or in cases with atypical (WHO grade II) or anaplastic meningiomas, adjuvant radiotherapy plays a crucial role in managing recurrence risk. Several studies have highlighted the efficacy of postoperative radiotherapy in reducing tumor regrowth, particularly in patients who underwent subtotal resection. For example, Mariniello et al. [3] reported that the addition of radiotherapy in patients with residual tumors after surgery significantly mitigated recurrence, with radiotherapy effectively controlling residual tumor growth in many cases. Similarly, Freeman et al. [16] found that postoperative radiotherapy was essential in managing high-risk cases and preventing early recurrence in patients with subtotal resections.

Neoadjuvant radiotherapy, although less commonly used in meningiomas, is an area of emerging interest, particularly in cases where tumor size or invasion into critical structures makes a complete surgical resection less likely. Neoadjuvant radiotherapy or radiosurgery may offer the benefit of reducing tumor size or slowing its progression before surgery, thus facilitating a more conservative and safer surgical approach. Gamma knife radiosurgery, in particular, has been employed in cases of small, well-circumscribed SOMs or for residual tumors post-surgery. Studies by Kong et al. [9] and Lekovic et al. [13] reported that patients who underwent radiosurgery following subtotal resection or for recurrent disease had favorable outcomes, with limited tumor progression and improved local control.

Discussion

SOMs are complex tumors that infiltrate both bone and soft tissues around the orbit, causing significant functional and cosmetic issues, including proptosis and vision loss. Surgical management now prioritizes symptom relief, focusing on reducing proptosis and restoring vision rather than radical resection. While complete resection is often limited by tumor invasion into critical areas like the cavernous sinus and superior orbital fissure, tailored surgical strategies can still achieve significant improvements in both function and appearance, with acceptable rates of complications and recurrence [27]. As demonstrated in this systematic review, managing SOMs requires a nuanced balance between achieving maximal resection and preserving critical functions, particularly vision and ocular motility.

The extent of surgical resection remains the most significant factor in determining long-term outcomes for patients with SOMs. GTR, involving complete removal of both the hyperostotic bone and soft tissue components, has been consistently shown to reduce recurrence rates. Studies included in this review highlight that GTR is associated with lower recurrence rates, often less than 20%, while STR leads to significantly higher recurrence rates, frequently exceeding 40%. However, achieving GTR in SOMs is often constrained by the proximity of the tumor to critical neurovascular structures.

Optic canal invasion, in particular, poses a major obstacle to complete resection. Involvement of the optic canal not only complicates surgical access but also significantly increases the risk of postoperative visual deficits. Studies by Mariniello et al. [3] and Freeman et al. [16] underscore the critical role of optic canal decompression in preserving vision in these patients, although the risk of recurrence remains high if tumor remnants are left behind. This balance between minimizing recurrence and avoiding vision loss requires precise surgical planning and experience, particularly when dealing with complex orbital and skull base anatomy.

Minimally invasive surgical techniques, such as the endoscopic transorbital approach, have emerged as potential alternatives to traditional fronto-temporal craniotomies, offering reduced morbidity and improved cosmetic outcomes. As demonstrated by Kong et al. [9], these approaches may allow for adequate tumor debulking while minimizing the extent of bony removal and thereby reducing complications such as CSF leaks and cranial nerve deficits. However, while initial results are promising, the long-term impact of these approaches on recurrence rates remains unclear due to the relatively short follow-up periods in the available studies. Further research is needed to establish whether these minimally invasive techniques can achieve recurrence rates comparable to those of more traditional approaches while maintaining functional outcomes.

Adjuvant radiotherapy plays a crucial role in the management of SOMs, particularly in cases where GTR cannot be achieved or in the presence of atypical (WHO grade II) or anaplastic meningiomas. Multiple studies included in this review, such as those by Mariniello et al. [3] and Freeman et al. [16], demonstrate that postoperative radiotherapy significantly reduces the risk of tumor regrowth, particularly in patients who undergo STR. This finding underscores the importance of a multidisciplinary approach in the treatment of SOMs, where radiotherapy serves as an essential adjunct to surgical resection, particularly in cases where complete resection is not feasible. Neoadjuvant radiotherapy, though less commonly employed, is an area of growing interest, particularly in cases where tumor size or invasion into critical structures makes GTR less likely. By reducing tumor volume preoperatively, neoadjuvant therapy may facilitate a safer and more conservative surgical approach, potentially reducing the risk of postoperative morbidity without significantly increasing the likelihood of recurrence. Gamma knife radiosurgery and proton therapy have also been employed in select cases of recurrent disease, demonstrating favorable outcomes in terms of local control and limited tumor progression [3,16].

While reducing recurrence remains a primary goal, functional outcomes-particularly in relation to vision-are equally critical in the management of SOMs. As this review illustrates, early surgical intervention, particularly with optic canal decompression, is strongly associated with better visual outcomes. Studies by Young et al. [15] and Sandalcioglu et al. [6] emphasize that patients presenting with severe preoperative visual deficits are less likely to experience significant postoperative improvement, highlighting the importance of early diagnosis and treatment. Complication rates, however, vary significantly depending on the extent of resection and the surgical approach employed. Common complications include cranial nerve deficits, CSF leaks, and infections, with newer endoscopic techniques appearing to reduce these risks. Nevertheless, long-term follow-up data on complications associated with minimally invasive techniques are still limited, and more research is needed to fully understand their safety profile.

This review has highlighted several important factors that influence outcomes in the management of SOMs. However, it is important to recognize the limitations inherent in the included studies. The heterogeneity of study designs, surgical techniques, and follow-up durations makes direct comparison of results challenging. Moreover, many studies rely on small sample sizes and retrospective data, limiting the generalizability of their findings. Future research should focus on prospective studies with longer follow-up periods to better evaluate the long-term efficacy of different surgical and adjuvant treatment modalities.

As surgical techniques continue to evolve, particularly with the advent of minimally invasive approaches, and as the role of radiotherapy becomes more clearly defined, the management of spheno-orbital meningiomas will continue to improve. A multidisciplinary approach, combining expert surgical resection with tailored adjuvant therapies, remains the cornerstone of effective treatment, offering the best chance for both long-term tumor control and preservation of function.

## Conclusions

SOMs present a unique challenge due to their complex location, proximity to critical neurovascular structures, and significant impact on both function and aesthetics. While gross total resection remains the gold standard for minimizing recurrence, it is often limited by the risk of neurological damage. Subtotal resection, combined with adjuvant therapies like radiotherapy, can offer a viable alternative, particularly for cases with critical structure involvement. Minimally invasive techniques show promise in reducing morbidity, though long-term data is still needed. The balance between achieving optimal tumor control and preserving function underscores the importance of a multidisciplinary approach tailored to each patient’s specific presentation. Future research should focus on refining surgical techniques and understanding the long-term outcomes of multimodal treatments to further improve both recurrence rates and patient quality of life.
